# A New Take on an Old Remedy: Generating Antibodies against Multidrug-Resistant Gram-Negative Bacteria in a Postantibiotic World

**DOI:** 10.1128/mSphere.00397-17

**Published:** 2017-10-04

**Authors:** Michael P. Motley, Bettina C. Fries

**Affiliations:** a Division of Infectious Diseases, Department of Medicine, Stony Brook University, Stony Brook, New York, USA; b Department of Molecular Genetics and Microbiology, Stony Brook University, Stony Brook, New York, USA; Albert Einstein College of Medicine

**Keywords:** *Acinetobacter*, *Enterobacteriaceae*, *Escherichia coli*, *Klebsiella*, ST258, MAb therapy, monoclonal antibodies, multidrug resistance

## Abstract

With the problem of multidrug-resistant Gram-negative pathogens becoming increasingly dire, new strategies are needed to protect and treat infected patients. Though abandoned in the past, monoclonal antibody therapy against Gram-negative bacteria remains a potential solution and has potential advantages over the broad-spectrum antibiotics they were once replaced by. This Perspective reviews the prospect of utilizing monoclonal antibody therapy against these pathogens, as well as the challenges of doing so and the current therapy targets under investigation.

## Abstract (precis)

With the problem of multidrug-resistant Gram-negative pathogens becoming increasingly dire, new strategies are needed to protect and treat infected patients. Though abandoned in the past, monoclonal antibody therapy against Gram-negative bacteria remains a potential solution and has potential advantages over the broad-spectrum antibiotics they were once replaced by.

## PERSPECTIVE

Multidrug-resistant (MDR) Gram-negative bacteria, including *Acinetobacter baumannii*, *Pseudomonas aeruginosa*, and carbapenem-resistant *Enterobacteriaceae* (CRE) (comprised of *Escherichia coli*, *Enterobacter* species, and *Klebsiella pneumoniae*), have been deemed urgent threats by the CDC. These pathogens commonly carry plasmids conveying resistance to multiple antibiotics, with some encoding carbapenemases. Carbapenemases deactivate a historically potent class of antibiotics that constitute the last line of defense against resistant organisms. Though carbapenemase genes have existed prior to 1985 when imipenem, the first FDA-approved carbapenem, was licensed, their prevalence has been promoted by the use of antibiotics. With limited antimicrobial agents available to fight them, these bacteria persist within their host with little challenge and cause serious disease. One example, the highly prevalent carbapenem-resistant *Klebsiella pneumoniae* (CR-*Kp*) sequence type 258 (ST258) clone, which possesses the *bla*_KPC_ carbapenemase gene, has been implicated with mortality rates of more than 40% (1). Less than a dozen new antibiotics have been approved since the new millennium, and some, including avibactam, have already selected for resistant clones (2). Additionally, only a few of the antibiotics currently in clinical development are directed against Gram-negative pathogens. Though antibiotic stewardship and studies of resistance spread are critical to stymie the spread of this pathogen, new therapies to fight already existent pathogens are warranted. Monoclonal antibodies (MAbs) are one such therapy, and they have been given increasing attention in recent years, given their specificity, limited risk of resistance development, and ability to work synergistically with antibiotics. In this Perspective, we will discuss the promise of, challenges with, and current attempts at utilizing MAbs in combating the growing threat of carbapenem-resistant Gram-negative bacteria.

## PROMISE

Immunotherapy, specifically serum therapy, was introduced as an antimicrobial treatment in the early 1900s. Although successful against several types of infections, the problems of allergic reactions, variable efficacy between lots, and limited spectrum led to its replacement in the 1930s by antibiotics, which were safer and capable of treating multiple pathogens. Over the past 50 years, however, immunotherapy has been advanced by the innovations of hybridoma technology, phage display platform, and protein engineering, leading to the development of therapeutic MAbs with improved safety and purity (3). Currently, at least 60 MAbs are approved for use in the United States (for the most updated list, refer to http://www.antibodysociety.org/news/approved-antibodies/ or use “mab” to search at Drugs@FDA for FDA-approved drugs [https://www.accessdata.fda.gov/scripts/cder/daf/]), a testament to the safety and efficacy of these treatments in a variety of settings. Still, most of these MAbs have been developed to treat malignancies and rheumatological diseases, with only four of these MAbs licensed for infectious diseases.

Antibodies are an effective therapeutic because of their specificity. Rather than binding to universal bacterial targets, most MAbs can bind to targets that are specific to the invading pathogen. This characteristic, which previously discouraged pharmaceutical companies due to lack of broad coverage, is also advantageous considering insights that wholesale disruption of the microbiota through broad-spectrum drugs can contribute to dysbiosis and diseases such as *Clostridium difficile* colitis. In addition, targeting of external virulence features such as poly-*N*-acetylglucosamine (PNAG) may actually create selective pressures for these bacteria to become less virulent (4).

Advantages of administering MAbs over active vaccination against MDR Gram-negative bacteria exist as well. First, MAb infusions are the only option for patients who lack the immunocompetence capable of mounting their own antibody response to vaccines, or active immunization, and these patients are the patients who most commonly manifest symptomatic infections with nosocomial pathogens such as CRE (1). Second, the administration of MAbs to select diagnosed patients, rather than targeting epitopes through large vaccination programs, could also limit selection pressures against these epitopes and preserve the efficacy of the target long term. Third, advances in engineering of MAbs have allowed researchers to create immunologic molecules with improved tissue penetration, enhanced recruitment of immune effectors, multiple variable regions with different specificities, and even the ability to assist with the precise delivery of other drugs that would be toxic systemically (3, 5). Last, some MAbs have been shown to act synergistically with antibiotics, improving their antimicrobial effect (5). These features highlight the promise of utilizing MAbs as unique alternatives to active vaccination and standard antibiotics once optimal targets against pathogens are identified.

## CHALLENGE

Several failed clinical trials for anti-infective biologics despite many preclinical successes warrant a better understanding of the true challenges in antibody design against bacterial pathogens (Table 1). One major hurdle to developing antibodies to combat bacteria is the lack of animal models that accurately model human infection. Because much of an antibody’s efficacy is dependent on its ability to coordinate with the host immune system rather than its ability to restrict growth in a test tube like antibiotics, MAbs must be studied preclinically within animal models. However, many current infection models employ unreasonably high inoculant amounts, laboratory-passaged standard strains, specific inbred mice, and unnatural routes of infection. Additionally, the pathogenicity of bacteria differs from species to species; in the case of CR-*Kp* ST258, strains that cause serious complicated infections in humans are rapidly cleared in mice and rats (6). These poor representatives of disease can distract researchers through preclinical successes that ultimately fail to translate into phase II results, or conversely, discourage antibody candidates that although unsuccessful in animals may be successful in humans.

**TABLE 1:** Known antibacterial monoclonal antibodies and related immunogic molecules that have entered clinical testing

Bacterial species	Gram stain	Drug	Type	Target	Indication	Company	Phase	Status
*Bacillus anthracis*	Gram positive	Obiltoxaximab (Anthim, ETI-204)	Humanized IgG1	Protective antigen (toxin)	Inhalational anthrax	Elusys	IV	FDA approved in 2016. In phase IV
	Gram positive	Thravixa, AVP-21D9	Human IgG1	Protective antigen (toxin)	Inhalational anthrax	Emergent	I	Development suspended. Successful phase I trial in 2011
	Gram positive	Valortim, MDX-1303	Human IgG1	Protective antigen (toxin)	Inhalational anthrax	PharmAthene	I	Development suspended. Successful phase I trial in 2011
	Gram positive	Raxibacumab (Abthrax)	Human IgG1	Protective antigen (toxin)	Inhalational anthrax	HGS/GSK	IV	FDA approved in 2012. Beginning phase IV
*Clostridium botulinum*	Gram positive	XOMA 3ab	Mix of 3 humanized IgG1	Botulinum neurotoxin type B (toxin)	Botulism	XOMA/NIAID	I	Development suspended. Successful phase I trial in 2013
	Gram positive	NTM-1632	Mix of 3 humanized IgG1	Botulinum neurotoxin type B (toxin)	Botulism	NIAID	I	In development. Completed phase I trial in 2017
*Clostridium difficile*	Gram positive	Actoxumab (CDA-1, MDX-066, MK-3415)	Human IgG1	*C. difficile* toxin A (toxin)	Colitis	Merck	III	Development discontinued. Failed to show additional efficacy with bezlotoxumab in phase III trial in 2015
	Gram positive	Bezlotoxumab (Zinplava, CDB-1, MDX-1388, MK-6072)	Human IgG1	*C. difficile* toxin B (toxin)	Colitis	Merck	III	FDA approved in 2016. In phase III for pediatric use
*Escherichia coli*	Gram negative	Edobacumab (XOMEN-E5)	Mouse IgM	LPS lipid A (exopolysaccharide/endotoxin)	Sepsis	XOMA	III	Development discontinued. Successful phase III trial in 1991. FDA approval withheld and failed in later studies
	Gram negative	Nebacumab (centoxin, HA-1A)	Human IgM	LPS lipid A (exopolysaccharide/endotoxin)	Sepsis	Centocor	III	Development discontinued. Successful phase III trial in 1991, failed in later studies
	Gram negative	T88	Human IgM	LPS lipid A (exopolysaccharide/endotoxin)	Sepsis	Chiron	III	Development discontinued. Failed phase III trial in 1995
STEC^a^	Gram negative	Shiga toxin MAbs, cαStx1 and -2	Mix of 2 humanized IgG1	*E. coli* Stx1 and Stx2 (toxins)	Bloody diarrhea in children	Taro (Thallion)	II	In development. Successful phase I trial in 2013
*Pseudomonas aeruginosa*	Gram negative	Aerucin	Human IgG1	*P. aeruginosa* alginate (exopolysaccharide)	Pneumonia	Aridis	II	In development. Open clinical trial
	Gram negative	Panobacumab (Aerumab, AR-101, KBPA-101)	Human IgM	*P. aeruginosa* LPS O11 (exopolysaccharide)	Pneumonia	Aridis (Kenta)	II	In development. Successful phase IIa trial in 2009
	Gram negative	KB001	Human PEGylated Fab	*P. aeruginosa* PcrV (secretion system)	Chronic infection in CF patients^b^	KaloBios	II	Development discontinued. Failed phase II trial in 2015
	Gram negative	MEDI3902	Bispecific human IgG1	*P. aeruginosa* PcrV (secretion system) and Psl (exopolysaccharide)	Ventilator pneumonia prevention	MedImmune	II	In development. Open clinical trial
*Staphylococcus aureus*	Gram positive	Salvecin, AR-301, KBSA-301	Human IgG1	*S. aureus* alpha-hemolysin (toxin)	Pneumonia	Aridis (Kenta)	II	In development. Completed phase II trial 2016
	Gram positive	ASN100 (ASN-1 and ASN-2 mix)	Mix of 2 human IgG1	*S. aureus* alpha-hemolysin, HlgAB, HlgCB, LukED, LukSF, and LukGH (toxins)	Ventilator pneumonia prevention	Arsanis	II	In development. Open clinical trial
	Gram positive	Tefibazumab (Aurexis)	Humanized IgG1	*S. aureus* ClfA (virulence protein)	Bacteremia, CF pneumonia	Bristol-Myers Squibb (Inhibitex)	II	Development discontinued. Failed phase II trial in 2006
	Gram positive	MEDI4893	Human IgG1 modified	*S. aureus* alpha-hemolysin (toxin)	Pneumonia	Astra Zeneca (MedImmune)	II	In development. Open clinical trial
	Gram positive	514G3	Human IgG3	*S. aureus protein A* (virulence protein)	Bacteremia	Xbiotech	II	In development. Completed phase II trial in 2017
	Gram positive	Pagibaximab (BSYX-A110)	Chimeric IgG1	Lipoteichoic acid (exopolysaccharide)	Sepsis in low-birth-weight infants	Biosynexus	III	Development discontinued. Failed phase III trial in 2011
	Gram positive	Aurograb	scFv	GrfA (lipoprotein)	Staphylococcal infection	NeuTec Pharma/Novartis	III	Development discontinued. Failed phase III trial in 2006
Multiple species	Gram positive and negative	F598	Human IgG1	Poly-*N*-acetylglucosamine (exopolysaccharide)	Multiple diseases	Alopexx	II	In development. Open clinical trial

a STEC, Shiga toxin-producing *Escherichia coli*.

b CF, cystic fibrosis.

Another major barrier of MAb development is antigenic heterogeneity of pathogens in the clinic. Recent expansion of mass sequencing has shown the extensive microevolution and heterogeneity of clinical strains and questions the generalizability of conclusions derived from research done only with standard laboratory strains (7). Protein epitopes of *E. coli* and *K. pneumoniae* have been shown to be highly conserved, but such conservation exists because these epitopes are concealed by layers of highly variable polysaccharides, including the O antigen found within lipopolysaccharide (LPS) and capsular polysaccharide (CPS), also known as K antigen (7). This variety necessitates either cross-reacting MAbs, cocktails of MAbs, or rapid accurate diagnosis prior to administration. The ability of broad-spectrum antibiotics to be used empirically in sepsis and against a plethora of pathogenic bacteria was a primary reason why antibody therapies were not developed in the first place, as broader indications consequently reach larger markets.

The structure of antibodies themselves presents some challenges as well. Unlike small-molecule drugs that bind individual targets, MAbs are large proteins with two—or more if engineered—binding moieties. Differences in the backbones of antibodies of differing isotypes and subclasses have been shown to affect not only downstream functions but also binding avidity by limiting the conformations of these moieties (8). More work must be performed to determine which isotypes and subclasses may improve binding and effector functions.

Fortunately, however, the large size of MAbs does not necessarily limit access to the site of the infection. Although sepsis can develop from primary septicemia, sepsis more often originates from deep-seated infections such as in the lung, kidney, or abdominal cavity in cases of *Enterobacteriaceae* infection. Experimental data with MAbs specific for staphylococcal enterotoxin B demonstrates that MAbs bind to their target in an abscess deeply seated within tissues, suggesting that like leukocytes, MAbs can home into these entrenched infections (9). In addition, ample data from murine studies indicate that intravenously administered MAbs can reach therapeutic levels in lung tissue, and even cross the blood-brain barrier in specific circumstances (10, 11). Furthermore, the recently FDA-approved bezlotoxumab (Zinplava; Merck) against *C. difficile* toxin has enlightened our understanding of the potential efficacy of MAbs directed against gut-colonizing pathogens. The demonstration that MAbs reach the colon after systemic administration (12), combined with data showing the role of the neonatal F_c_ receptor in the transcellular shuttling of MAb-bound pathogens (13), show the ability of MAbs to limit infections in the intestinal lumen. Murine experiments with MAbs that target *K. pneumoniae* further support this notion, as these data demonstrated that antibiotic-induced dissemination of *K. pneumoniae* that colonize the gut can be significantly lessened with systemic *Klebsiella*-specific MAb treatment (14).

Last, because infections can progress faster than malignancies or immunologic diseases, the precise timing of when to administer MAbs to treat infections also becomes a challenge. Those MAbs that have been shown to prevent disease could be administered prophylactically as passive immunization to at-risk patients. For example, patients at long-term-care facilities colonized with CR-*Kp* who are admitted to the hospital for antibiotic therapy are significantly more at risk of developing CR-*Kp* infection (15). With MAb half-lives usually lasting 2 to 3 weeks, a single dose upon admission to the emergency room could be sufficient to protect these patients for the duration of a typical hospital stay. However, at present, MAb therapy is expensive, and the cost-effectiveness of prophylactic treatment against infections has been debated (16). MAbs given as postinfection therapy would be cheaper and could be combined with traditional antibiotics to further lessen symptoms or improve outcomes. The concern for this modality though would be that for immunocompromised patients with poor immune systems, late treatment might be ineffective if the infection has progressed too far. However, median times between infection and death are reported to be more than a week for some MDR pathogens, suggesting that these infections progress slowly and may be susceptible to treatment even after diagnosis (1, 17). While significant challenges remain in discovering their proper targets and optimizing their effects, MAbs are generally safe, and with further technological advances to reduce cost, their pharmacokinetic properties can allow them to be developed into efficacious therapeutics.

## TARGETS

Given the increasing scarcity of effective antibiotics previously mentioned, anti-infective antibodies capable of mediating MDR Gram-negative infections could be valuable as a treatment or prophylactic against nosocomial and iatrogenic infections in high-risk patients. Although in past years anti-infective MAbs have advanced into clinical testing, there are only nine MAbs targeting Gram-negative organisms, and development of several of these have been discontinued (Table 1). Nevertheless, recent developments in targeting these bacteria, from new takes on traditional targets to relatively new ones, provide promise for the future. We discuss these targets below.

### Lipopolysaccharide.

Targeting the quintessential macromolecule of Gram-negative bacteria and mediator of sepsis was one of the first approaches to immunotherapy against *Enterobacteriaceae* bloodstream infections. Since the 1960s, numerous groups have attempted to utilize active immunization, antiserum therapy, and intravenous immunoglobulin administration against LPS to reduce mortality in patients with septic Gram-negative bacterial infections, as LPS was shown by numerous studies to be highly immunogenic (reviewed in reference 18). The most successful of these targeted the core lipid A and oligosaccharide epitopes that were more conserved to circumvent the challenge presented by the extensive variation of the outer O-antigen polysaccharide. However, although these candidate vaccines and antibody therapies demonstrated success in eliciting immune responses and limiting mortality in preclinical models and even small human sepsis trials, none of the three anti-LPS MAbs that underwent clinical trials ultimately succeeded. It is possible that the location of the internal LPS regions the antibodies attempted to target compromised their ability to reach their target. Additionally, IgM molecules tend to possess shorter half-lives and greater steric hindrance than IgG molecules, which are more often used in MAb therapy.

Interestingly, recent findings from studying anti-LPS antibodies in *Klebsiella pneumoniae* suggest that anti-LPS antibodies that neutralize endotoxin may prevent the activation of host immunity necessary to ultimately clear the infection (19). Those antibodies which promoted opsonophagocytosis but not LPS neutralization were shown to be potentially superior (19). Accordingly, most screens for potent anti-infective antibodies today commonly incorporate opsonophagocytic assays (4–6, 14, 20–22), providing encouragement that future efforts to target LPS will be successful.

Novel attempts to target LPS have been productive, with panobacumab, an anti-pseudomonal O11 LPS MAb currently in clinical development (23), and other MAb candidates in preclinical development. One of these new candidates takes advantage of LPS homology among strains of CR-*Kp* (6). Nearly all CR-*Kp* strains present in the United States belong to the ST258 clonal group, 83% of which share the O-antigen d-galactan III (24). A new humanized antibody against the antigen has been shown to inhibit endotoxin-mediated Toll-like receptor 4 (TLR4) activation better than polymyxin B *in vitro*, as well as improve survival in endotoxemia-susceptible mice (6). Additionally, while the antibody improved *in vitro* opsonophagocytic killing and serum killing only of those ST258 strains that expressed d-galactan III LPS, it did cross-react against strains of different CPS types common to CR-*Kp* (mentioned below). Therefore, while not a panacea against all LPS-carrying bacteria, this candidate may at the very least provide a target against those specific *Klebsiella* strains that antibiotics cannot combat.

### Capsular polysaccharide.

Targeting capsular polysaccharide (CPS), which constitutes an important bacterial defense that prevents phagocytosis by macrophages and neutrophils, has been one of the most successful strategies in the design of antibacterial immunotherapy. Specifically, efficacy of this approach has been demonstrated through their use in active immunization against *Streptococcus pneumoniae*, *Neisseria meningitidis*, *Haemophilus influenzae*, and even the *Enterobacteriaceae Salmonella* serovar Typhi. While CPS also possesses abundant antigenic variability, a property well studied in *E. coli* and *K. pneumoniae*, studies have found that some CPS vaccines can induce immune responses that cross-react between serotypes (25). Consequently, the use of MAbs that bind protective CPS antigens against *Enterobacteriaceae* have been attempted, including the administration of intravenous immunoglobulin derived from donors immunized with a 24-valent anti-*Klebsiella* CPS vaccine (26). Though the trial failed to show significant differences in mortality, it did show a small reduction in type-specific *Klebsiella* species infections when administered to 1,400 intensive care patients.

The promise of anti-CPS MAbs to combat MDR Gram-negative bacteria is further encouraged by studies that show that the capsules of some clinical isolates of species such as *Acinetobacter baumannii* and CR-*Kp* have greater homology than across the species as a whole. An IgM against the K1 capsule of *A. baumannii*, called 13D8, was shown to promote neutrophil-mediated killing and rescue from soft tissue infections of K1 capsule-positive strains, which comprise 13% of sampled *Acinetobacter* strains (27). Additionally, despite the fact that strains within the CR-*Kp* ST258 clonal group vary with respect to traditional K serotypes (7), their variation with respect to more-conserved genes within the CPS locus is significantly lower. Full sequencing of more than 80 CR*-Kp* clinical strains in the United States found that the majority of strains fit into two or three different lineage clades and that strains within these clades generally carry only one or two distinct alleles of the *wzi* gene, which helps affix CPS to the surfaces of bacteria (28, 29). Although the *wzi* gene is only one of many genes responsible for CPS synthesis, our own work has found that it can predict cross-agglutination by MAbs: an IgM developed from immunization of mice with two ST258 CPS from two different *wzi* strains agglutinated all ST258 strains of the same capsule type as well as several unrelated capsule types (29). The data suggest that development of cross-reactive anticapsular antibodies may be highly effective against those drug-resistant species with high clonality. However, not all carbapenem-resistant Gram-negative species have a single dominant clone like *K. pneumoniae*, and if they do, not much work has examined the variability of CPS genes of these clones. More epidemiological studies assessing these qualities are necessary to determine if these species can also be targeted by capsule-specific MAb therapy.

Although no anticapsular antibodies against MDR Gram-negative pathogens have entered clinical trials, our laboratory has already shown as a proof of principle that such antibodies can be used to protect against invasive disease (14, 27). We recently generated antibodies against the CPS of the hypervirulent but carbapenem-sensitive K1 serotype of *K. pneumoniae* (14). This K1-specific MAb not only promotes opsonophagocytic killing and complement deposition but most importantly, conveys protection against invasive disease in a murine colonization model where dissemination from the gut is induced by treatment with antibiotics.

### Conserved exopolysaccharides.

More broadly conserved exopolysaccharides have also been pursued as immunotherapy targets against MDR Gram-negative bacteria. One target, the exopolysaccharide Psl found in *P. aeruginosa*, was found from screening a phage display library derived from patients infected with *Pseudomonas* against whole bacteria (21). The antibody generated from the screen promoted opsonophagocytic killing, inhibited epithelial binding of 12 *Pseudomonas* clinical isolates of different O serotypes, and prevented mortality in mice. The binding region of the antibody has since been incorporated into the bispecific antibody MEDI3902, which combined with the anticytotoxic properties granted by the anti-PcrV domain further improved survival in murine infection models, and additionally showed synergistic activity with antibiotics (5). This bispecific antibody and another anti-*Pseudomonas* antibody targeting the polysaccharide alginate, is currently undergoing clinical testing (ClinicalTrials.gov identifiers NCT02696902 and NCT03027609, respectively).

Other exopolysaccharides are even more conserved—the surface carbohydrate PNAG is expressed in a variety of prokaryotic and eukaryotic pathogens (30). The anti-PNAG MAb F598 demonstrated efficacy in protecting mice infected intraperitoneally with different species of CRE, as well as mice infected intravenously with *bla*_KPC_-expressing CR-*Kp* (4). As PNAG is found not only in MDR Gram-negative bacteria but also in other prominent pathogens such as *Staphylococcus aureus* and *Mycobacterium tuberculosis*, the antibody could have the advantage of being used as broad-spectrum MAb therapy while blood cultures and susceptibility tests are being run. However, care must be taken if such an antibody is implemented; like currently used antibiotics, these antibodies may have unintentional effects on the microbiome, including selection for other pathogens or variants. Specifically, while it has been suggested that loss of PNAG by selective pressure can reduce the virulence of some organisms (4), modifications to PNAG that reduce MAb binding may still retain virulence. Additionally, the natural evolution of an anticapsular humoral response in patients colonized with CRE should be studied further, as anti-CPS antibodies could potentially compete with PNAG antibodies in human serum (31). Nevertheless, murine data on PNAG-specific antibodies are promising, and clinical trials have begun recruiting to test these antibodies in patients with urinary tract infections caused by *Neisseria gonorrhoeae* (NCT03222401).

### Pilus formation proteins.

Pili are crucial to the role of multidrug-resistant Gram-negative bacteria in adhesion and biofilm formation, providing an opportunity to target exposed epitopes (32). Antibodies against these pili may serve to reduce urinary tract infections that can be caused both by *E. coli* and *K. pneumoniae*. For example, *mrkA*, a gene encoding the fimbrial protein MrkA that contributes to the type III secretion system, has been strongly implicated in urogenital adhesion and biofilm formation, and was found in all 69 clinical isolates tested, although actual expression of the protein was less frequent and the multilocus sequence type was not determined for these isolates (32). This antigen was conjectured to be a potential target against CR-*Kp* ST258 by some groups (33). Coincidentally however, antibodies against MrkA were also discovered through a blind screen utilizing a naive phage display library as well as hybridoma development against live *K. pneumoniae* lacking capsular polysaccharide and O antigen (20). These MAbs promoted opsonophagocytic killing independent of LPS serotype, reduced biofilm formation *in vitro*, and limited organ burden and mortality in murine infection models (20, 22). These findings, as well as the success of MEDI3902 (5), demonstrate not only the possibility of utilizing pili as a target for antibody development but also the worth of phage display-based screening platforms in discovering targets as well.

Although proteins are likely to be more-conserved targets to preserve function, conformational changes in these proteins may alter the binding site of an antibody and prevent attachment and execution of effects. Indeed, a study of those MrkA antibodies showed that they recognize not a peptide sequence, but rather epitopes generated by folding or multimer formation (22). Conversely, however, antibodies can be engineered to stabilize less-virulent conformations. One such antibody binds the active site of *E. coli* FimH, a component of type I adhesive pili, but also allosterically converts the protein to a conformation that prevents binding to mannose (34). The antibody has subsequently been shown to limit the ability to *E. coli* to develop biofilms, and it has also been shown to reduce the severity of bladder infection in mice with urinary tract infections.

### Extracellular vesicle components and other proteins.

Extracellular vesicles are released from Gram-negative bacteria, and their vesicles commonly contain important virulence factors such as toxins. As a result, vesicles have been used to identify potential targets for immunotherapy (35). Vesicles derived from *K. pneumoniae* were shown to induce an immunogenic response with an increase in species-specific antibodies. While the immunity to the vesicles was cell mediated as well as humoral, serum transfer of immunized mice to naive mice infected with *K. pneumoniae* showed mild mortality protection (35). For CR-*Kp* ST258, numerous proteins that are overrepresented on the surfaces of membrane vesicles have been identified (33). Targeting these proteins may allow for rapid neutralization of these vesicles and limiting selection of resistant mutants while reducing negative effects on healthy bacteria.

Additionally, other targets identified by the same proteomic approaches may also inspire future work on developing antibodies against MDR Gram-negative organisms. Highly conserved proteins like OmpA, for example, have been shown to be effective and attractive targets in other Gram-negative bacteria (36). These approaches will hopefully diversify the search for anti-Gram-negative antibodies and permit the discovery of an effective solution should the other approaches fail.

## CONCLUSION

The use of monoclonal antibodies to treat or prevent sepsis caused by *Escherichia coli*, *Klebsiella pneumoniae*, *Acinetobacter baumannii*, and other MDR Gram-negative bacteria is a rational goal that is increasingly becoming a realistic approach. While previous efforts have resulted in failure, bright new candidates continue to emerge, as we both discover and attempt to utilize new targets and improve efforts to try old targets. Furthermore, the entire field of anti-infective antibodies has grown, as we begin to understand the roles of isotype and structural components in specificity and function. While challenges exist, we expect to see anti-infective antibodies playing a role against antibiotic-resistant organisms and other pathogens in the future.
